# Spectacle Design Preferences among Chinese Primary and Secondary Students and Their Parents: A Qualitative and Quantitative Study

**DOI:** 10.1371/journal.pone.0088857

**Published:** 2014-03-03

**Authors:** Zhongqiang Zhou, Maja Kecman, Tingting Chen, Tianyu Liu, Ling Jin, Shangji Chen, Qianyun Chen, Mingguang He, Josh Silver, Bruce Moore, Nathan Congdon

**Affiliations:** 1 State Key Laboratory of Ophthalmology and Division of Preventive Ophthalmology, Zhongshan Ophthalmic Center, Sun Yat-sen University, Guangzhou, Guangdong, China; 2 Center for Vision in the Developing World, St Catherine's College, Oxford, Oxfordshire, United Kingdom; 3 Guangming Eye Hospital, Yangjiang, Guangdong, China; 4 New England College of Optometry, Boston, Massachusetts, United States of America; 5 ORBIS International, New York, New York, United States of America; Zhongshan Ophthalmic Center, China

## Abstract

**Purpose:**

To identify the specific characteristics making glasses designs, particularly those compatible with adjustable glasses, more or less appealing to Chinese children and their parents.

**Patients and Methods:**

Primary and secondary school children from urban and rural China with < = −1.00 diopters of bilateral myopia and their parents ranked four conventional-style frames identified by local optical shops as popular versus four child-specific frames compatible with adjustable spectacles. Scores based on the proportion of maximum possible ranking were computed for each style. Selected children and their parents also participated in Focus Groups (FGs) discussing spectacle design preference. Recordings were transcribed and coded by two independents reviewers using NVivo software.

**Results:**

Among 136 urban primary school children (age range 9–11 years), 290 rural secondary school children (11–17 years) and 16 parents, all adjustable-style frames (scores on 0–100 scale 25.7–62.4) were ranked behind all conventional frames (63.0–87.5). For eight FGs including 12 primary children, 26 secondary children and 16 parents, average kappa values for NVivo coding were 0.81 (students) and 0.70 (parents). All groups agreed that the key changes to make adjustable designs more attractive were altering the round lenses to rectangular or oval shapes and adding curved earpieces for more stable wear. The thick frames of the adjustable designs were considered stylish, and children indicated they would wear them if the lens shape were modified.

**Conclusions:**

Current adjustable lens designs are unattractive to Chinese children and their parents, though this study identified specific modifications which would make them more appealing.

## Introduction

Uncorrected refractive error remains the leading cause of vision loss between 6/18 and 3/60, and the second-leading cause of blindness, in the world [1]–[2].Though refractive error may be safely and reliably corrected with inexpensive spectacles, a very substantial burden of visual disability persists, particularly in developing areas [3]–[4], due to a lack of refractive services and the frequency of inaccurate spectacles that do not adequately correct vision [5]. Uncorrected refractive error is associated with significant self-reported visual disability among children [6], which is readily remediable when glasses are provided [7].

The lack of well-trained refractionists in settings of limited resources is an important barriers to correction of refractive error, and it has been demonstrated that increasing access to refractionists in rural areas leads to improved vision [8]. Self-refraction by children with adjustable glasses has been suggested as an alternative or adjunct to training of further refractionists where capacity is currently limited. Recent studies [9]–[10] have suggested that children can achieve vision of > = 6/7.5 in >90% of cases by self-refraction with adjustable spectacles, and that accuracy when compared to subjective refraction by an experienced optometrist may equal or exceed that of automated refraction without cycloplegia, another technique commonly used in areas where trained refractionists are rare.

Though alternative designs exist, the spectacles used in most published trials on children to date have fluid-filled lenses whose power is adjusted by adjusting the volume of fluid and thus the thickness of the lens. This approach imposes certain design constraints on the glasses, including the fact that the lenses themselves are relatively thick, and lens shapes other than circular are somewhat challenging to implement. Though few published data are available, it has been suggested that these designs may be unappealing to children [11], hindering the widespread use of adjustable glasses in correcting vision, as opposed to simply as refractive devices. Dis-satisfaction with cosmetic appearance and fear of being teased have been identified as barriers to spectacle wear among children [12]–[13], though little evidence exists in the peer-reviewed literature about design features which might make glasses more or less appealing to children of different ages.

The WEAR (Wearability and Evaluation of Adjustable Refraction) Study is designed to assess various aspects of the practical usefulness of adjustable spectacles both as refractive devices and as corrective eyewear among Chinese children. The current report includes both a qualitative (Focus Group) and quantitative component, with the aim of:

- Comparing rankings of popular conventional frames with new frame designs for adjustable spectacles among myopic children of various ages and their parents
- Identifying the key characteristics that make glasses designs, in particular those compatible with adjustable glasses, more or less appealing

## Materials and Methods

The protocol for this study was approved in full by the Institutional Review Boards of the Zhongshan Ophthalmic Center (ZOC), Sun Yat-sen University (Guangzhou, China).Permission was also received from the local Board of Education. Informed written consent was obtained from > = one parent of all participants. The consent form was distributed to every child one week prior to examination. Only those who returned signed consent forms were enrolled in the study. This consent procedure was approved by the ZOC IRB. The principles of the Declaration of Helsinki were followed throughout.

### Selection of participants for quantitative (questionnaire-based) segment

Children of consenting parents from all 18 classes in grades 7 and 8 (generally 12–15 years old) at two rural junior high schools in Yangjiang, Guangdong Province, southern China, underwent visual acuity (VA) screening from September to October, 2012. This area has previously been identified as having high prevalence of refractive error but low rates of spectacle wear among children requiring glasses [3], and the accuracy of spectacles prescribed in this region of China has been demonstrated to be poor [5]. Thus, this area is representative of the kind of setting in which adjustable spectacles might be used. Current spectacle wear was not a criterion for participation in the study.

VA was tested separately for each eye at a distance of 4 meters using Early Treatment Diabetic Retinopathy Study (ETDRS) charts (Precision Vision, La Salle, IL, USA) in a well-lighted, indoor area of the school. Children with presenting VA< = 6/12 in either eye received automated refraction (Topcon KR 8900, Tokyo, Japan) without cycloplegia, and those with < = −1.00D of myopic refractive error in both eyes were eligible to participate, as were their parents.

Additionally, children in grade 4 (generally 9–10 years old) in 8 urban primary schools in Guangzhou city participating in an ongoing clinical trial (the Guangzhou Outdoor Activity Longitudinal (GOAL) study, NCT00848900) underwent assessment of VA and automated refraction with cycloplegia (Topcon KR 8900, Tokyo, Japan) using the identical protocol between November and December 2012. Children with uncorrected VA < = 6/12 in either eye and myopic refractive error < = −1.00D bilaterally were eligible, as were their parents. Urban children were selected in order to determine whether their preferences would differ from those of rural children and their families.

### Implementation of questionnaires

**Figure 1:**
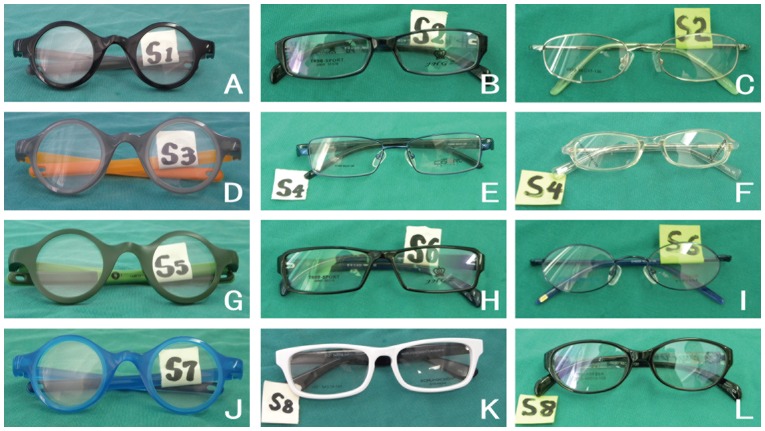
Pictures of 12 frames. Among them, B, E, H and K are conventional frames for rural secondary school children and their parents, C, F, I and L are conventional frames for urban primary school children and their parents, A, D, G, and J are frames designed to accommodate adjustable spectacles, which were the same for both groups.

Primary and secondary school children and their parents were all asked independently to rank a group of eight labeled spectacle frames displayed for them on a table, according to which they found most attractive for themselves (or their children) to wear. The eight frames consisted of two inter-mixed sets: four conventional styles provided by large spectacle shops in Yangjiang (the rural setting) and Guangzhou (the urban setting) as being their best sellers for children, and four frames (differing only by color) designed for use with adjustable spectacles made specifically for children, and provided by the Centre for Vision in the Developing World, St Catherine's College, Oxford. (**Figure 1**) All of the frames were displayed without lenses, and mirrors were supplied so that children could examine themselves wearing the glasses. Each child examined the frames by him or herself, whereas parents reviewed them in small groups with no children present. All rankings were recorded by the subjects themselves on supplied forms and without discussion. A total of 12 frame styles were ranked (four conventional frames for rural secondary school children and their parents, four conventional frames for urban primary school children and their parents, and four frames designed to accommodate adjustable spectacles, which were the same for both groups), but each subject was presented only with eight styles to rank.

### Selection of participants for the focus groups (FGs)

Two key constituencies, myopic children and their parents, took part in separate FGs in Yangjiang (n = 4 for myopic secondary school children, n = 2 for their parents) and Guangzhou city (n = 2 for myopic primary school children, n = 1 for their parents). Children were selected at random from among participants in the questionnaire portion of the study, and thus met identical criteria (presenting VA< = 6/12 in either eye and myopic refractive error < = −1.00D bilaterally). The intent was to include 5–10 subjects in each group, so as to maximize potential for interaction while minimizing the possibility of some subjects not having the opportunity to speak. Among the total of nine FGs, recording equipment malfunctioned for one group of secondary school children in Yangjiang whose data could not be analyzed, leaving 8 groups consisting of 12 primary children, 26 secondary children and 16 parents with data.

### Implementation of FGs

Separate scripts for children's and parents' FGS, consisting of 6–7 open-ended questions concerning preferences for spectacle design were drafted by the principal investigator, an experienced FG researcher (NC), and reviewed and revised by all authors. (**Table 1**) Two facilitators (ZZ and CT) received intensive training from the PI prior to implementation of the FGs. They ran each FG together, first giving a brief general introduction to the FG process, and then leading discussions based on the scripted questions. The eight spectacle frames described above were supplied as props for each of the FGs. FGs lasted an average of 40 minutes each, were carried out in Cantonese and/or Mandarin between October 12 and December 05, 2012, and were recorded digitally and subsequently transcribed into written Chinese by a trained team of three transcriptionists.

**Table 1:** Questions asked of children and parents participating in Focus Groups on the appearance and design of spectacles.

Participants	Questions
Children and parents	How does your family decide which glasses you will buy? What role do you play? Your parents (children)? Others?
Children and parents	Where do you go to buy your glasses? How long do you spend deciding which glasses to buy?
Children and parents	What/who influences what kind of glasses you get for yourself (your child)?
Children and parents	How important is the appearance of the glasses you buy in deciding whether you (your child) will wear them?
Children and parents	What kinds of things might make glasses look better or worse on a child?
Children and parents	What kinds of things do you think your parents (children) feel make glasses look better or worse on a child?
Children and parents	(For each of the colors and styles of glasses): What do you like about the way these look? What don't you like? How would you change them to make them more attractive? Would you be likely to wear them the way they look now? What if they were modified the way you suggest?
Children and parents	When and where might you be more likely to wear your glasses?
Children	How do you feel about the way glasses make you look?
Children	What kind of glasses do you think look best on you? Have people ever commented about your glasses to you? Who? What kinds of things have they said?

• How does your family decide which glasses you will buy? What role do you play? Your parents (children)? Others?

• Where do you go to buy your glasses? How long do you spend deciding which glasses to buy?

• What/who influences what kind of glasses you get for yourself (your child)?

• How important is the appearance of the glasses you buy in deciding whether you (your child) will wear them?

• What kinds of things might make glasses look better or worse on a child?

• What kinds of things do you think your parents (children) feel make glasses look better or worse on a child?

• (For each of the colors and styles of glasses): What do you like about the way these look? What don't you like? How would you change them to make them more attractive? Would you be likely to wear them the way they look now? What if they were modified the way you suggest?

• When and where might you be more likely to wear your glasses?

• (For children only).

• How do you feel about the way glasses make you look?

• What kind of glasses do you think look best on you? Have people ever commented about your glasses to you? Who? What kinds of things have they said?

### Statistical Methods

Participants' rankings for the 8 frame styles were analyzed using Stata 10.0 (Stata Corp, College Station, TX, USA) in the following fashion: 8 points were assigned for each participant who ranked a particular style highest, 7 for a second-place ranking, and so forth, with the lowest ranking receiving a score of 1. A total score for each style was calculated as the sum of points awarded across all participants, which was then divided by the maximum possible score to facilitate comparison across groups of different sizes. Thus, the maximum score would be 100% and the minimum score 1/8 = 12.5%.

Two investigators (TL and ZZ) coded all interview transcripts independently using NVivo 8.0 (QSR, Inc., Melbourne, Australia), after reviewing all 8 transcripts and reaching agreement on a coding scheme. The final scheme was entered as “Tree Nodes,” which were identical for both coders. The inter-rater coding reliability (Cohen's Kappa) was calculated using the “Coding Comparison” query provided by NVivo, as percentage agreement of passages coded to the appropriate nodes. By convention, a Kappa greater than 0.7 is considered acceptable inter-rater reliability [14].

## Results

### Questionnaire-based ranking of frame styles

A total of 426 children with presenting VA< = 6/12 in either eye and myopic refractive error < = −1.00D bilaterally were enrolled, including136 urban primary school students (age range 9–11 years, 49.3% male) and 290 rural secondary school students (age range 11–17 years, 48.3% male). The spherical equivalent refractive error (RE) of the right eye of the primary and secondary students were −2.47±1.23 and −2.42±0.08 diopters (D), respectively. (**Table 2**)

**Table 2:** Basic Demographic Characteristics and Refractive Error of Participants.

Characteristics	Subjects
	Number (%)
**Age (years)**	
Primary students	
9	83 (61.0)
10	50 (36.8)
11	3 (2.21)
Total	136 (100.0)
Secondary students	
12–13	87 (30.0)
14	113 (39.0)
15	70 (24.1)
16–17	20 (6.89)
Total	290 (100.0)
**Sex, N (%)**	
Primary students	
Male	67 (49.3)
Female	69 (50.7)
Total	136 (100.0)
Secondary students	
Male	140 (48.3)
Female	150 (51.7)
Total	290 (100.0)
**Spherical equivalent refractive error**	(Mean ± SD)
Primary students	−2.47±1.23†
Secondary students	−2.42±0.08‡

† Auto- refraction with cycloplegia.

‡ Auto- refraction without cycloplegia.

All 426 children (100%) completed the glasses frame style ranking questionnaires. The percentage of the theoretical maximum possible score achieved by four adjustable glasses-specific frame designs among 136 primary school children ranged from 39.7 to 62.4, while the scores for conventional frames fell from 63.0 to 68.7 (**Table 3**). Among secondary school children the ranges for adjustable-style and conventional frames were 25.7–49.9 and 65.8–80.2 respectively (**Table 3**). All 16 parents completed ranking forms as well, with scores for adjustable-style and conventional frames ranging from 31.3 to 45.3 and 64.8 to 87.5 respectively (**Table 4**).

**Table 3:** Children's ranking of 8 frame styles (See Figure 1 for pictures of the frames).

Frame Style	Ranking*										
	#1	#2	#3	#4	#5	#6	#7	#8	Total Respondents	Total Score	% of Maximum Possible Score
**Primary students**											
**Standard frames**											
Style 2	24	23	25	12	10	17	12	13	136	691	**63.5**
Style 4	26	28	21	26	12	7	9	7	136	754	**69.3**
Style 6	23	18	23	21	14	12	15	10	136	685	**63.0**
Style 8	33	20	17	25	18	6	9	8	136	747	**68.7**
**Frames designed for adjustable eyeglasses**											
Style 1	3	12	9	9	23	29	29	22	136	466	**42.8**
Style 3	5	5	8	13	18	31	23	33	136	432	**39.7**
Style 5	0	11	15	12	18	19	25	36	136	442	**40.6**
Style 7	22	19	18	18	23	15	14	7	136	679	**62.4**
**Secondary students**											
**Standard frames**											
Style 2	56	88	83	27	17	14	3	2	290	1815	**78.2**
Style 4	31	40	65	79	34	14	8	19	290	1526	**65.8**
Style 6	84	84	62	30	5	12	10	3	290	1861	**80.2**
Style 8	102	42	41	69	17	8	8	3	290	1812	**78.1**
**Frames designed for adjustable eyeglasses**											
Style 1	10	10	18	41	118	53	24	16	290	1158	**49.9**
Style 3	1	5	5	16	27	68	102	66	290	735	**31.7**
Style 5	1	5	5	12	23	31	65	148	290	596	**25.7**
Style 7	4	16	11	16	49	91	70	33	290	932	**40.2**

*#1 indicates the highest ranking receiving a score of 8 and #8, the lowest ranking receiving a score 1.

A total score for each frame style was calculated as the sum of points awarded across all participants, which was then divided by the maximum possible score in order to facilitate comparison across groups of different sizes. The maximum possible score was equal to total respondents times 8.

**Table 4:** Parents' ranking of 8 frame styles (See Figure 1 for pictures of the frames).

Frame style	Ranking*										
	#1	#2	#3	#4	#5	#6	#7	#8	Total respondents	Total score	% of maximum possible score
**Standard frames (ranked by primary school parents)**											
Style 2	1	1	1	0	0	1	1	0	5	26	**65.0**
Style 4	0	1	2	1	1	0	0	0	5	28	**70.0**
Style 6	1	1	1	2	0	0	0	0	5	31	**77.5**
Style 8	3	1	0	0	1	0	0	0	5	35	**87.5**
**Standard frames (ranked by secondary school parents)**											
Style 2	0	8	2	0	0	0	1	0	11	70	**79.5**
Style 4	2	0	2	5	0	1	0	1	11	57	**64.8**
Style 6	6	1	2	0	0	2	0	0	11	73	**83.0**
Style 8	1	1	4	3	0	0	2	0	11	58	**65.9**
**Frames designed for adjustable eyeglasses (ranked by all primary and secondary school parents)**											
Style 1	0	1	0	2	7	3	1	2	16	58	**45.3**
Style 3	0	0	1	1	2	5	4	3	16	45	**35.2**
Style 5	0	1	0	0	3	3	3	6	16	40	**31.3**
Style 7	2	0	1	2	2	1	4	4	16	55	**43.0**

*#1 indicates the highest ranking receiving a score of 8 and #8, the lowest ranking receiving a score 1.

A total score for each frame style was calculated as the sum of points awarded across all participants, which was then divided by the maximum possible score in order to facilitate comparison across groups of different sizes. The maximum possible score was equal to total respondents times 8.

### FGs: participants and inter-reviewer agreement on coding

Participants in the FGs included 12 primary children (age range 9–11 years, 7 [58.3%] girls), 26 secondary children (age range 11–16 years, 14 [53.8%] girls) and 16 of their parents (5 [31.2%] fathers and 11 [68.8%] mothers; 5 [31.2%] parents of primary school children, 11 [68.85] parents of secondary school children). After independent review by two coders of transcripts for the 8 FGs, kappa values for the student and parent groups were 0.81 and 0.70, respectively. Table 5 shows the response nodes among children for topics related to spectacle wear, as an example.

**Table 5:** Coded responses of children in focus groups.

Topic area	Response nodes
**Family decision-maker when buying glasses**	Primary school: Sometimes this is the child, more often the parents, both will listen to each other's suggestions in making the decision.
	Secondary school: Children make the decision. Only a few of them are concern about their parents' suggestions.
**Whether children have role models**	Primary and Secondary: Yes, but most of these role models don't wear glasses, and kids think their role models look better without glasses.
**Whether role models' appearance with glasses influence your use**	Primary: No
	Secondary: A little, children like glasses with big black frames and think these are fashionable, as these are worn by some media personalities.
**Source for refraction and glasses purchase**	Primary: From hospital eye departments, some go to private optical shops.
	Secondary: Most purchase glasses at optical shops
**Factors in choosing glasses**	Primary: Comfortable (nose pad), light, attractive shape (rectangular or oval), color, material: plastic, not metal
	Secondary: Color, fashion, price, material, the pattern on the frame, comfortable
**Importance of the appearance of glasses**	Primary: Important in choosing glasses
	Secondary: Important, the first consideration when buying glasses
**Factors that might make glasses look better on a child**	Primary: Color (black, blue, pink, purple); shape (square, oval, big frame); material (plastic, light); some like frame without patterns, some like patterns like flowers or rabbits; curved temples
	Secondary: Color (black, blue, purple, pink); some student like a mixed-color frame, such as black and white; rectangle or oval frame, full frame; material (most prefer plastic, only a few like metal); slim, curved temples, the pattern on the frame
**When and where glasses are worn**	Primary: When necessary, such as reading the blackboard, watching TV, doing homework
	Secondary: Some wear glasses almost all day, but some only wear them when necessary, such as in class, or when watching TV
**Reasons for not wearing glasses**	Primary: No need, easy to lose or damage while playing sports.
	Secondary: Feel glasses are inconvenient, afraid wearing glasses will worsen myopia
**Feelings about your appearance when you wear glasses**	Primary: No idea, or a bit better
	Secondary: Some are used to their appearance with glasses, and think it looks good; some still don't like their appearance with glasses
**Comments made by others on your wearing glasses**	Primary: Some negative comments, some have been laughed at, but most are not concerned by this.
	Secondary: Some negative comments and teasing, but not of great concern; some people think children with glasses look well-educated

### FGs: Frame preferences and appearance of glasses

Comfort, lightness, material (plastic was generally preferred over metal) and shape were important to both primary and secondary school children in their evaluation of glasses design, though secondary school children emphasized that appearance was their primary concern in choosing glasses. Both groups of children expressed a preference for square or oval frames rather than round; thick, dark-colored frames were particularly popular among secondary children. Preferred colors among both primary and secondary children were blue, black, purple and pink, the latter among girls. Feelings (positive and negative) about their own appearance while wearing glasses were stronger among the older than younger children.

Parents were more concerned about price and durability of frames for their primary school children, but acknowledged the importance of appearance to secondary school children. Parents' preferences for plastic frames, square and oval shapes and preferred colors were very similar to children, including thick, dark frames for older children.

When asked how they would change the frames designed for adjustable glasses to make them more attractive, there was strong agreement between primary and secondary children and parents: the most important feature requiring alteration was the round shape, which should be made rectangular or oval. Children also favored adding designs to the temples (“rabbits,” “a McDonald's logo,” “leopard stripes.”) There was general agreement among parents and children that the earpiece of the temples should be curved downward to hold the glasses in place, rather than straight as in current designs. The thick frames of the adjustable-style glasses were deemed attractive or not an issue. Primary and secondary children generally agreed that if the shape of the glasses were altered and the temples curved, they would be willing to wear them.

### FGs: Decision-makers and sources for glasses purchase

Children and parents agreed that secondary children principally made their own choices about glasses, with little input from parents. Primary children indicated that they shared decision-making, though their parents felt that they (parents) were principally responsible for selection. Parents and children agreed that they were much more likely to go to the hospital (where cycloplegia can legally be carried out) to purchase spectacles for younger children, while older children and their parents both indicated that their glasses were purchased primarily at free-standing shops (without medical staff present).

### FGs: Influences on choice and use of glasses

Both primary and secondary children acknowledged having role models, though they and their parents agreed that few of these wore glasses. One influence on principally older children from media figures was the preference for thick, dark frames. Neither older nor younger children expressed much concern about teasing due to glasses wear, though they admitted this sometimes happened; older children also mentioned that some observers thought children wearing glasses appeared more-educated. Appearance of glasses frames was important to both older and younger children in deciding whether to wear glasses. Though both older and younger children expressed concern that glasses might be inconvenient or easily lost, especially during sports, older children were more likely to wear them regularly. Younger children were more likely to wear glasses only when needed (in school, when watching TV), though some older children described such intermittent wear as well. Parents were more likely to describe their primary school children as seldom wearing their glasses, and older children as wearing them when needed.

## Discussion

The quantitative portion of this study highlighted the fact that current child-specific frame designs for adjustable glasses are unpopular with primary and secondary school-aged urban and rural children and their parents. FG results pinpointed the round lens shape as the feature most widely considered to require revision in order to make adjustable spectacle-compatible designs more attractive. The fluid-filled lens design of these adjustable glasses requires that the frame be relatively thick, and modifying the frame to accommodate a rectangular or oval shape would necessitate additional material being added. It is thus encouraging to note that thick frames were generally considered stylish by both parents and children, especially among older students more concerned with frame appearance, apparently because this type of frame is commonly worn by Chinese media figures. It was also encouraging that older and younger children generally agreed during the FGs that they would be willing to wear adjustable-lens compatible glasses from a design standpoint if their suggested modifications were carried out.

FG data also highlighted differences in patterns of spectacle acquisition between families of children of different ages: parents appeared to take a more active role in frame selection for younger children, while both parents and children agreed that older children were permitted more independence in frame selection. This greater leeway in selection of preferred designs is important in view of evidence that children are more likely to wear spectacles when they are given a choice of styles [13]. FG data also revealed that parents were more likely to purchase spectacles for younger children in a hospital setting, while glasses for older children were more commonly obtained from free-standing optical shops. This presumably reflects the fact that only medically-licensed personnel are permitted to administer topical cycloplegia in China, which for practical purposes mean that most independent shops, staffed principally by non-medical practitioners, are prohibited from performing cycloplegic refraction. Automated refraction without cycloplegia is often used, which is known to result in significant inaccuracies in younger children due to instrument accommodation [15]–[16].

Findings in the current study that children, particularly older children with stronger design preferences, prefer thicker, dark-colored frames, and that oval and square lenses are preferred over round ones, are potentially important to persons and organizations developing refraction programs targeting the large, highly-myopic populations of southern China. This is particularly true if frames will be brought in from outside the region rather than purchased locally, where providers are likely to be more aware of local preferences. Subjective preference for different spectacle designs is likely to vary greatly by region, age and gender, among other factors. Nonetheless, in view of ample evidence that appearance of glasses significantly affects children's willingness to wear them [12]–[13], there is clearly a need for research in this area. Despite this, our review of the PubMed database in August 2013 for articles in English using the words “spectacle,” “glasses,” “design,” “attractive,” “style,” “preference,” “appearance” and “children” failed to identify any articles concerned with specific design factors and their impact on wear or acceptability to children or adolescents. The relatively small number of articles identified focused exclusively on adults [17]–[19].

Strengths of the current study include enrolment of younger and older children from rural and urban areas and their parents, and having utilized a combination of qualitative and quantitative techniques to more thoroughly elucidate design preferences and specific suggestions to improve the acceptability of adjustable glasses frame design. The reliability of major findings regarding preferred designs is reinforced by the high degree of agreement across quantitative and qualitative techniques and stakeholder groups. Weaknesses include the fact that these highly-subjective design preferences may only with caution be applied to other settings. Given the high prevalence of uncorrected refractive error in southern China, and the large population of children there, information about this specific population is important in and of itself. Further, we hope that publication of this paper may encourage investigators working in other areas to carry out similar work. Secondly, it must be acknowledged that preferred styles of spectacle frames may change over time, meaning that work such as this may need to be updated regularly. Still, a better understanding of current preferences may allow researchers to elucidate more general trends and constant factors. Finally, frames were displayed to children without functioning lenses, as the goal of the study was to assess frame design. It may be that children would respond differently to glasses with the lenses in place, though with current technology, the lens design is not amenable to change, and so we elected not to study this aspect.

We hope to incorporate these findings into future designs of child-specific adjustable-power glasses in order to maximize their acceptability to target populations, and thus their impact in reducing the burden of uncorrected refractive error in China.
